# From HIV diagnosis to treatment: evaluation of a referral system to promote and monitor access to antiretroviral therapy in rural Tanzania

**DOI:** 10.1186/1758-2652-12-31

**Published:** 2009-11-11

**Authors:** Ray Nsigaye, Alison Wringe, Maria Roura, Samuel Kalluvya, Mark Urassa, Joanna Busza, Basia Zaba

**Affiliations:** 1 Tazama Project, National Institute of Medical Research, Mwanza, Tanzania; 2 Centre for Population Studies, London School of Hygiene and Tropical Medicine, London, UK; 3 HIV/AIDS Unit, Bugando University College of Health Sciences, Mwanza, Tanzania

## Abstract

**Background:**

Individuals diagnosed with HIV in developing countries are not always successfully linked to onward treatment services, resulting in missed opportunities for timely initiation of antiretroviral therapy, or prophylaxis for opportunistic infections. In collaboration with local stakeholders, we designed and assessed a referral system to link persons diagnosed at a voluntary counselling and testing (VCT) clinic in a rural district in northern Tanzania with a government-run HIV treatment clinic in a nearby city.

**Methods:**

Two-part referral forms, with unique matching numbers on each side were implemented to facilitate access to the HIV clinic, and were subsequently reconciled to monitor the proportion of diagnosed clients who registered for these services, stratified by sex and referral period. Delays between referral and registration at the HIV clinic were calculated, and lists of non-attendees were generated to facilitate tracing among those who had given prior consent for follow up.

Transportation allowances and a "community escort" from a local home-based care organization were introduced for patients attending the HIV clinic, with supportive counselling services provided by the VCT counsellors and home-based care volunteers. Focus group discussions and in-depth interviews were conducted with health care workers and patients to assess the acceptability of the referral procedures.

**Results:**

Referral uptake at the HIV clinic averaged 72% among men and 66% among women during the first three years of the national antiretroviral therapy (ART) programme, and gradually increased following the introduction of the transportation allowances and community escorts, but declined following a national VCT campaign. Most patients reported that the referral system facilitated their arrival at the HIV clinic, but expressed a desire for HIV treatment services to be in closer proximity to their homes. The referral forms proved to be an efficient and accepted method for assessing the effectiveness of the VCT clinic as an entry point for ART.

**Conclusion:**

The referral system reduced delays in seeking care, and enabled the monitoring of access to HIV treatment among diagnosed persons. Similar systems to monitor referral uptake and linkages between HIV services could be readily implemented in other settings.

## Background

HIV testing services have expanded rapidly in many developing countries in order to reach ambitious targets for antiretroviral therapy (ART) coverage [1]. However, the potential for testing services to act as a gateway to HIV treatment can be met only if individuals diagnosed with HIV are subsequently linked to onward care and treatment services in a timely manner. Delays in registering at HIV treatment clinic services following an HIV diagnosis can lead to late initiation of prophylactic treatment against opportunistic infections or ART, potentially resulting in poorer prognoses for patients and an additional clinical burden on overstretched health services [2].

In many settings, HIV services are currently organized such that there are a great deal more HIV testing points than treatment clinics, with diagnosed persons from several testing sites theoretically linking to each HIV treatment centre. The World Health Organization has repeatedly acknowledged the importance of strengthening links between HIV testing and HIV treatment sites, stating that explicit mechanisms are necessary to promote referral to onward medical and psychosocial support for those testing positive [3,4]. Simple methods for monitoring onward referral rates are particularly important in the context of provider-initiated testing and counselling and prevention of mother to child transmission services, and for monitoring subsequent access to HIV-related services, thus ensuring that ethical concerns about routine testing strategies are addressed [5].

Although international guidelines have emphasized that improved referral mechanisms are essential for promoting and monitoring entry to HIV services [6,7], few referral tools have been developed, and as a result there is a paucity of data on the number of persons who are successfully linked with treatment services following an HIV diagnosis. Nevertheless, emerging evidence from projects involved in referring HIV-diagnosed persons to HIV clinics in Tanzania has suggested rates of referral uptake as low as 14%, representing missed opportunities for timely HIV care and ART initiation [8]. Similarly low rates of referral uptake at HIV clinics have been noted following diagnoses made at voluntary counselling and testing (VCT) services that are provided through mobile outreach clinics [9]. In this case, diagnoses are made conveniently close to patients' homes, but the HIV treatment clinic may be far away, and after the mobile VCT service has moved on, there is no-one left for patients to consult. Indeed, in terms of accessing treatment following an HIV diagnosis, transportation costs have been identified as an important barrier to reaching these services [10,11], with allowances rarely provided at the point of diagnosis.

The effectiveness of referral systems between the various echelons of the health system in sub-Saharan African countries has been explored in relation to other conditions, with several studies focusing on reasons for non-adherence to referral advice or analyses of health systems inefficiencies when patients are treated at higher level facilities than necessary [12-15]. However, few studies have reported rates of referral uptake, with the exception of two studies of "down referrals" of HIV and TB patients from hospitals to health centres, which indicated an overall attrition rate between facilities of approximately 30% [16,17]. The most commonly cited reasons for poorly functioning referral systems include systemic factors, such as inadequate training, poor quality referral letters or a lack of feedback between facilities [13,16,17]. Furthermore, patient-level factors, including economic or opportunity costs and preferences for, or proximity to, certain facilities, have also been shown to influence uptake of referral advice [12,13,15].

Until effective referral systems for HIV treatment are more widely implemented, it is difficult to evaluate how effective or equitable different HIV testing sites are in terms of enabling access to onward care and treatment services [18], or to devise locally relevant, low-cost interventions to improve referral uptake. In order to maximize the benefits of HIV testing, simple and robust referral systems are therefore needed to promote timely access to treatment services for infected patients, enable delays and equity in the uptake of referral appointments to be monitored, and facilitate communication between different HIV service providers so that low rates of referral uptake can be documented and acted upon.

In this context, we piloted a new method for monitoring referral rates between a VCT clinic in a rural area in north-west Tanzania and a government-run HIV treatment clinic in a zonal referral hospital, 20 km away in Mwanza City. We also documented the role of transport allowances and a volunteer escort from a local home-based care (HBC) organization on rates of referral uptake. This paper describes the implementation of this referral system and reports on the lessons learned that enabled us to improve access to HIV treatment in the community, and that can be readily adopted elsewhere.

## Methods

### Study setting

In Tanzania, the national AIDS control programme began providing free ART in public sector referral hospitals, and subsequently rolled out treatment to district hospitals and health centres [19]. HIV testing is provided by more than 500 static and mobile VCT clinics and through "opt-out" or provider-initiated testing and counselling, which has been implemented in health care facilities and antenatal clinics since 2007 [6,7].

Monitoring and evaluation (M&E) of the national ART rollout is coordinated by the Tanzanian Commission for AIDS, focusing on routine data collection for key indicators, including numbers of individuals initiating ART. In selected areas of the country, such as the TAZAMA study site, specialized M&E research is being undertaken to document the uptake and demographic impact of ART in the context of a long-term HIV cohort study [20].

This study, located in Kisesa ward in the rural north-west part of the country, collects longitudinal demographic and serological surveillance data, providing a rich background against which M&E of HIV service uptake can occur. An integral aspect of the project's activities is to pilot data collection tools that can be adopted nationally for monitoring access to HIV services.

### Development of the referral system

The process of designing the referral system was led by researchers from the Tanzanian National Institute of Medical Research, and included consultations with stakeholders involved in referring diagnosed HIV-infected clients from VCT to the HIV treatment clinic. These included VCT counsellors, HIV clinic staff, representatives from a local HBC programme (the Lutheran Church-run "Tumaini", which supported HIV-infected persons in the area), and the national AIDS control programme. Other local projects referring HIV-positive persons to the HIV treatment clinic (such as a microbicide development study) also participated to avoid duplicating efforts and developing parallel systems which might increase the workload of clinic staff.

The aims of the referral system as defined during these consultations were: (1) to ensure that HIV-positive VCT clients were formally directed and linked to HIV treatment services; and (2) to monitor the proportion of HIV-positive VCT clients who were successfully linked to the HIV clinic, in order to assess the effectiveness of the VCT clinic as a "gatekeeper" to the HIV treatment programme.

The "Tumaini" programme, now operating under the name "Tunajali" provided home-based care in the area and conducted tracing visits among those of its clients who do not take up referral appointments. During the study period, two home-based care volunteers were recruited from each village and received a small monthly stipend of approximately US$8. From early 2006, Tumaini was also able to provide volunteer community escorts to accompany newly diagnosed patients from the local VCT centre to the HIV treatment clinic. Transportation allowances (approximately US$2 for a return trip) were also provided for patients to attend the HIV treatment clinic from early 2006 and were managed by the VCT counsellors.

### Development of the referral forms

The content and format of the forms that were used to facilitate and document referral rates were developed after reviewing existing referral forms from other African settings [21], and were piloted during 2005. The final version of the referral form consisted of two detachable parts, with matching, unique numbers on each side to facilitate subsequent reconciliation of the two parts.

One side included basic socio-demographic information about the referred person and the referral date, and was given to the patient to present at the HIV treatment clinic. This section was completed by registration nurses at the HIV clinic, and included the date of the patient's registration, allowing the delay between referral and registration to be calculated. By additionally recording the unique patient identifiers assigned by the HIV clinic on this section, referral data could be subsequently linked to the data recorded in patients' HIV clinic files.

The remaining part of the form also included the patient's socio-demographic information, and was retained by the referring party. The unique VCT identifier allocated by the counsellors for each patient was recorded on this slip to enable referral data to be subsequently linked to the VCT data. A template of the referral forms can be downloaded from http://www.tazamaproject.org. Referral slips were regularly collected from the VCT clinic and the HIV treatment clinic, and reconciled by a clinical research officer using a data-entry programme that generates standard anonymized monthly reports. All referral slips were stored in a locked cupboard to ensure patient confidentiality.

### Quantitative methods

Data were analysed using Stata 10 (StataCorp, College Station, Texas, USA). The proportion of diagnosed clients who were referred to the HIV treatment clinic and the proportion of referred patients that subsequently registered at the HIV treatment clinic were calculated, stratified by sex and time period. Delays in registering at the HIV treatment clinic following a referral were calculated by subtracting the date of registration from the date of referral. Cross tabulations and chi square tests were conducted to assess for associations between sex or time period and uptake of a referral or registration at the HIV treatment clinic.

### Qualitative methods

In order to explore the acceptability of the referral system, we conducted in-depth interviews with 11 health care workers involved in referring and receiving patients at the HIV treatment clinic 18 months after introducing the referral procedures. Within the context of a wider qualitative study exploring access to HIV services [22,23], we also conducted 42 in-depth interviews and four focus group discussions with referred patients to elicit their experiences of using the referral system. The focus group discussions and in-depth interviews were recorded with prior consent from participants and then transcribed, translated into English, and entered into NVIVO7 for analysis. A coding scheme was derived from the data by assigning codes to major concepts mentioned by the participants that were related to the referral scheme.

### Ethical approval

Ethical approval for the study was obtained from the Medical Research Coordinating Committee (Tanzania) and London School of Hygiene and Tropical Medicine (UK).

## Results

### Referral rates

The referral system between the VCT centre and HIV treatment clinic enabled us to monitor trends in the uptake of referral appointments and assess the effectiveness of the VCT service in linking diagnosed patients to available treatment services. Overall, we observed a high referral rate over the three-year period, with close to 100% of men and women receiving a referral following their diagnosis at VCT. High proportions of referred clients subsequently registered at the HIV clinic within six months of their referral, with no statistically significant difference in uptake rates between men and women (72%, 84/117 versus 66%, 153/232; p = 0.27).

**Figure 1:**
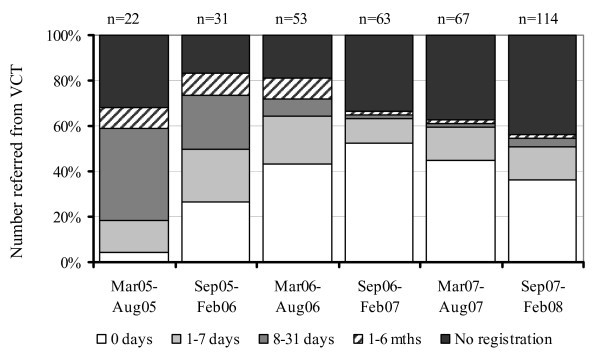
**Delays in registering at the HIV treatment clinic following referral from VCT**.

Over the three-year period, there was a steady increase in the overall number of HIV-infected persons who were referred, as well as the number who subsequently registered at the HIV clinic within six months of referral (from 22 to 114, and 15 to 64, respectively) (Figure 1). During the first 18 months of the referral programme, the proportion of patients who registered at the HIV treatment clinic within a week of their referral more than tripled from 18% to 64%, although the proportion who remained unregistered after six months never went lower than 17%.

Between September 2007 and February 2008, the number of clients who were referred increased by 70% compared with the preceding six months, coinciding with a national HIV testing campaign. However, over the same period, the proportion of referred persons who did not subsequently register at the HIV treatment clinic also increased by 7% compared with the preceding six months (Figure 1).

### Acceptability of the referral system

The characteristics of the participants in the qualitative study are shown in Table 1, and the main findings that emerged in relation to the referral system are summarized in Table 2.

**Table 1:** Characteristics of participants in the qualitative study

			*Interviewed*
**Activity**	**Variable**	**Category**	**n**

**In-depth interviews (patients)**	***Total***		***42***
	Sex	Male	14
		Female	28
	Age	15-24	2
		25-34	21
		35-44	16
		45+	3
	Period referred from VCT	Mar 05-Sep 05	17
		Sep 05-Feb 06	7
		Mar 06-Sep 06	18

**In-depth interviews (healthcare workers)**	***Total***		***11***
	Sex	Male	2
		Female	9
	Role	VCT counsellor	2
		HIV clinic staff	6
		HBC worker	3

**Focus group discussions (patients)**	***Total***		***46***
	Sex, area of residence and age	Men rural villages, all ages	6
		Women rural villages, all ages	11
		Men roadside villages, all ages	11
		Women roadside villages, all ages	18

**Table 2:** Summary of referral system activities and main emerging issues

Problem	Solutions	Implementer	Main issues
Financial constraints	Transport allowance	TAZAMA	• Helped many PLHIV attend the HIV clinic
			• Could become difficult for VCT counsellors to administer
			• Sometimes spent on items other than fare

Reaching the clinic	Escort	TUMAINI	• Facilitated initial access to the HIV clinic for many PLHIV, especially those not familiar with city environment
			• Heavy and possibly unsustainable workload for volunteer with an increasing number of patients
			• Difficulties in arranging convenient times for escort and patients to meet
	Referral forms	TAZAMA/BMC hospital	• Effective in facilitating access to the HIV clinic and enabling HIV clinic staff to identify Kisesa patients
			• Enabled low uptake of referral appointments to be identified and described
			• Enabled list of non-attendees to be generated for tracing by home-based care teams
			• Facilitated data exchange between VCT clinic & HIV clinic

Initiating clinic visits	Supportive counselling	VCT counsellors/TUMAINI	• Helped some patients overcome concerns about initiating HIV clinic visits
			• Supportive counselling from VCT counsellors was mostly accessed by residents living close by

PLHIV: People living with HIV

The provision of a transportation allowance was widely acknowledged by the patients to facilitate uptake of referral appointments at the HIV treatment clinic. As one woman explained, accessing this modest financial support enabled her to overcome the financial barriers to attending the clinic that she had been facing:

The problem that I had was about transport, that is what was troubling me. And at that time I didn't have money that I could pay for my fare... therefore I was not going there constantly.... But afterwards when I got a sponsor, they were giving me an allowance and I attended (adherence) training continuously ... [Roadside, woman, in-depth interview]

However, there were also a few reports of patients facing pressure from their families to spend the transport allowance on other items, including food, reflecting the precarious economic situation of some HIV patients. One HBC worker explained the competing priorities faced by some patients who had received transport money:

[They say]...without food I will die. So why not die tomorrow because I have no fare rather than today because I have no food. [HBC worker, in-depth interview]

High levels of alcohol dependency were reported in the study setting both by HIV patients and health care workers, and there were some reports that the transport allowance was used to purchase local alcohol instead of paying the bus fare.

These problems were generally overcome once a volunteer from the HBC programme became available to escort patients during their first visit to the HIV treatment clinic. Additional reported benefits of the escort included having someone on hand to provide encouragement, as well as physical support for those in poor health. The presence of an escort was seen to be particularly important for patients who were unfamiliar with the journey to Mwanza City, or who felt intimidated by attending a big hospital and could reduce the delay in taking up the referral appointment, as one of the HBC volunteers explained:

Right now what we do once he/she gets that green [referral] card, you go with him direct. At least during the first trip you take them to Bugando [hospital] there. After he/she arrives there they are comforted in their heart to see that it's so many people ... But during first trip you have to take him/her there. If you don't, he/she can take that green [referral] sheet and stay with it at home! [HBC worker, in-depth interview]

The provision of referral forms also played an important role in facilitating patients' entry into the hospital, or ensured that they were directed to the right clinic. Furthermore, patients from Kisesa were not required to repeat VCT on arrival at the HIV clinic in the hospital, because the referral forms included the signature of a recognized VCT provider and would be difficult to duplicate due to their design and green colour. Avoiding the need for these rural patients to repeat VCT at the hospital reduced the time that they needed to spend at the HIV clinic completing the registration requirements:

We receive patients at the reception there and talk with them. Patients coming from TAZAMA bearing those green referral forms we don't take them to VCT because we know that VCT done at Kisesa there is similar to the one done here at Bugando [hospital]. [HIV clinic staff, in-depth interview]

The referral forms were also perceived by the nurses to be a useful tool that aided the registration process because they included key background information about the patient. Finally, the referral forms acted as an indicator to the nurses of the general level of preparedness of the patients, particularly in terms of their knowledge about the clinic procedures and need for follow-up appointments:

Those green forms are good because they show his/her history, so even before we interview him/her we have got more of his/her information already ... It also helps to know the services they get there [from TAZAMA] ... we even know that the patient is willing. Other patients come to clinic without fare or whatever, so they are fearful. But for the patients coming from Kisesa we are sure that this one will follow our services. [HIV clinic staff, in-depth interview]

Although most patients reported that the referral system facilitated their initial access to HIV treatment, many expressed a desire for HIV treatment clinics to be in closer proximity to their homes:

So I request services to begin at the [health] centres in each area. It's easy to go, and perhaps we can succeed on this problem, to be known early and treated early before the infection advances. [Roadside, male, in-depth interview]

## Discussion

In this setting, we explored the extent to which a referral system could be used to promote access to an HIV treatment clinic among individuals diagnosed at VCT, and to monitor rates of referral uptake. Similar mechanisms for monitoring referral rates could be implemented in any sites linking HIV-infected individuals with prevention, care, treatment and support services. Although it may not be appropriate for all linked HIV services to analyse referral uptake data, special studies can be conducted using this system to monitor the effectiveness of different HIV testing services in promoting access to ART, and can provide important insights into the degree of equity in access that is being achieved.

Furthermore, in selected sites, such as Kisesa, where community-level HIV data are collected through regular surveys, these analyses can be extended to monitor the entire process of accessing and initiating HIV treatment at a population level, enabling much-needed local estimates of ART coverage relative to treatment need, disaggregated by sex, residence and age to be derived [24].

Additional qualitative research in this setting has shown that even when economic barriers, such as the cost of transport, are addressed, knowledge and psychosocial issues remain important barriers to accessing HIV services [22,23,25-27]; these include HIV-related stigma, lack of family support, and denial of illness, as well as use of alternative healers. The referral uptake data generated through this method enabled us to compile a list of persons who did not register for treatment services following referral. HBC volunteers then provided additional support to these patients in the form of further information about HIV infection and associated prevention and treatment options. These patients had given prior consent to such contacts during the VCT session and lived locally, thus helping to overcome some of the barriers to attending the HIV clinic for patients who delayed their initial clinic appointment.

Furthermore, by monitoring appointment uptake, we were able to observe variations in referral uptake in relation to the level of support services that were being provided. Initial increases in the proportions taking up their referral appointment within a week correspond with the introduction of the community escort and transportation allowances at the beginning of 2006.

The lower proportion accessing the HIV clinic following a diagnosis made during the national campaign suggests that increasing opportunities to learn one's status may not necessarily translate into effective access to HIV care and treatment, unless adequate resources for supportive counselling are also made available. In particular, the surge in the number of persons diagnosed during the last six months of the study period put pressure on the community escort scheme, such that it became difficult to offer this service to all referred patients during this period. It is also likely that the HIV testing campaign attracted individuals who were, on average, at an earlier stage of HIV infection compared with the population who actively sought VCT at the health centre, of whom a high proportion reported poor health as their reason for testing. This may have contributed to lower levels of motivation or readiness to attend the HIV treatment clinic among some persons who were diagnosed during the HIV testing campaign, partially explaining the lower overall referral uptake rates during this period.

The provision of a transportation allowance has been proposed as a strategy for improving access to HIV services, as well as to promote ART adherence and retention in care in several settings [11,28], and emerged as an important intervention in Kisesa in terms of facilitating regular attendance at the HIV treatment clinics. The cost of covering the return fare to the HIV treatment clinic in this setting was in the region of US$25-30 per patient per year, corresponding to a fraction of the total costs of providing medical treatment to HIV-infected patients, and is considered a sustainable use of programme funds by donor agencies, including the Global Fund for AIDS, TB and Malaria.

As such, the provision of transport fees should be viewed as an investment in terms of promoting timely registration for ART screening, which could result in earlier initiation of treatment and reduce the high mortality rates that have been widely observed among patients starting ART with very low CD4 counts [29,30]. Transportation allowances are also likely to facilitate adherence to treatment by delaying the need for second-line treatment, which cost around 10 times more than current generic first-line regimens.

Alternative strategies to donor-provided transportation fares should draw on lessons learned from programmes that have reported successes in using community cost-sharing or insurance schemes to cover transportation costs for referrals between primary and secondary level facilities [13,31]. Nevertheless, the longer-term solution needs to focus on bringing treatment services closer to local populations if barriers relating to the cost of reaching clinics are to be successfully addressed. Emerging evidence suggests that decentralization increases uptake of HIV treatment services and results in higher rates of retention in care [32,33]. This process of decentralization needs to be accompanied by interventions that address wider structural and social barriers that influence HIV clinic attendance, including poverty and stigma [22,23,26].

The involvement of key stakeholders throughout the design and implementation process led to high acceptance levels and satisfaction with referral monitoring procedures. Following this experience, other referral agencies linked to the same HIV clinic have adopted the same forms and are currently monitoring HIV treatment access [8]; similar systems are being piloted in other African countries. We have also used the same method to monitor referrals between VCT and the local HBC group. The next challenge is to encourage its adoption by other HIV testing services, including provider-initiated testing and counselling and those where subsequent referral uptake may be particularly low, such as mobile, door-to-door or other services outside a clinic environment, to ensure that national and international recommendations regarding strengthened linkages between testing and treatment sites are met, and that access to ART is improved.

The potential limitations of this study include the fact that referred individuals may have attended the HIV treatment clinic without their referral slip, or may have attended private providers, thus leading to an underestimate in the proportion of referred persons who subsequently accessed HIV care. In order to assess this, we cross checked registration books at the HIV treatment clinic to see if residents from the study area had enrolled for treatment having lost their slip, and found this to be the case for very few persons, whom we subsequently included in our uptake calculation.

Furthermore, in this setting, where the average income is approximately US$120 per year [34] and the average cost of a month of antiretroviral therapy drugs is in the region of US$25, it is unlikely that residents in the area were seeking private care. In terms of the qualitative data, we limited the potential for respondent bias by using trained fieldworkers, who established a rapport with study participants and explained the non-judgemental purpose of the study prior to commencing interviews and group activities.

## Conclusion

In conclusion, an HIV diagnosis is not always sufficient to ensure that infected persons are subsequently able to access HIV treatment, including ART. In this paper, we described the methods that we used to document delays in referral uptake at a hospital-based ART site following diagnosis in a rural VCT clinic, and the lessons learned through this process. The implementation of this simple referral system helped to assess the effectiveness of the VCT clinic as an entry point for HIV treatment and facilitated timely access to treatment for HIV-positive individuals in this rural area.

Similar systems to monitor referral uptake and linkages between HIV services could be readily implemented in other settings. A failure to strengthen referral procedures as HIV testing expands would be an unacceptable lost opportunity to ensure the highest of ethical standards and a commitment to promoting equitable access to life-prolonging antiretroviral drugs.

## Competing interests

The authors declare that they have no competing interests.

## Authors' contributions

RN was responsible for the overall management of the referral system, liaison with stakeholders, and the data collection and analysis. He co-wrote the first draft of the manuscript. AW contributed to the design of the referral system, co-wrote the first draft of the manuscript, and co-designed the qualitative study. MR co-designed the qualitative study, recruited and trained qualitative researchers, and provided advice on the qualitative work. SK contributed to the design and implementation of the referral system, and the design of the evaluation. MU, director of the whole cohort study, provided overall advice, facilitated the coordination between the stakeholders, and contributed to drafting the manuscript. JB provided technical support in designing the qualitative study. BZ, technical advisor for the whole cohort study, conceived the initial idea for the referral system, provided advice, and contributed to drafting the manuscript. All co-authors read and commented on the draft versions of the paper and participated in the editing process.
